# Icaritin greatly attenuates β‐amyloid‐induced toxicity in vivo

**DOI:** 10.1111/cns.14527

**Published:** 2023-11-21

**Authors:** Liangxian Li, Zaiwa Wei, Yafang Tang, Mingyue Jin, Hua Yao, Xia Li, Qinghua Li, Jie Tan, Bo Xiao

**Affiliations:** ^1^ Laboratory of Respiratory Disease Affiliated Hospital of Guilin Medical University Guilin China; ^2^ Guangxi Key Laboratory of Brain and Cognitive Neuroscience Guilin Medical University Guilin China; ^3^ Clinical Research Center for Neurological Diseases of Guangxi Province Affiliated Hospital of Guilin Medical University Guilin China; ^4^ Guangxi Engineering Research Center for Digital Medicine and Clinical Translation Affiliated Hospital of Guilin Medical University Guilin China; ^5^ The Key Laboratory of Respiratory Diseases Education Department of Guangxi Zhuang Autonomous Region Guilin China

**Keywords:** Alzheimer's disease, energy metabolism, Icaritin, mitochondria, oxidative stress

## Abstract

**Aims:**

The accumulation and deposition of β‐amyloid (Aβ) has always been considered a major pathological feature of Alzheimer's disease (AD). The latest and mainstream amyloid cascade hypothesis indicates that all the main pathological changes in AD are attributed to the accumulation of soluble Aβ. However, the exploration of therapeutic drugs for Aβ toxicity has progressed slowly. This study aims to investigate the protective effects of Icaritin on the Aβ‐induced *Drosophila* AD model and its possible mechanism.

**Methods:**

To identify the effects of Icaritin on AD, we constructed an excellent *Drosophila* AD model named Aβ_arc_ (arctic mutant Aβ_42_) *Drosophila*. Climbing ability, flight ability, and longevity were used to evaluate the effects of Icaritin on AD phenotypes. Aβ_arc_ was determined by immunostaining and ELISA. To identify the effects of Icaritin on oxidative stress, we performed the detection of ROS, hydrogen peroxide, MDA, SOD, catalase, GST, and Caspase‐3. To identify the effects of Icaritin on energy metabolism, we performed the detection of ATP and lactate. Transcriptome analysis and qRT‐PCR verifications were used to detect the genes directly involved in oxidative stress and energy metabolism. Mitochondrial structure and function were detected by an electron microscopy assay, a mitochondrial membrane potential assay, and a mitochondrial respiration assay.

**Results:**

We discovered that Icaritin almost completely rescues the climbing ability, flight ability, and longevity of Aβ_arc_ *Drosophila*. Aβ_arc_ was dramatically reduced by Icaritin treatment. We also found that Icaritin significantly reduces oxidative stress and greatly improves impaired energy metabolism. Importantly, transcriptome analysis and qRT‐PCR verifications showed that many key genes, directly involved in oxidative stress and energy metabolism, are restored by Icaritin. Next, we found that Icaritin perfectly restores the integrity of mitochondrial structure and function damaged by Aβ_arc_ toxicity.

**Conclusion:**

This study suggested that Icaritin is a potential drug to deal with the toxicity of Aβ_arc,_ at least partially realized by restoring the mitochondria/oxidative stress/energy metabolism axis, and holds potential for translation to human AD.

## Graphical abstract

Icaritin greatly rescues the phenotypes of the Aβ‐induced *Drosophila* AD model, and its possible mechanism is almost completely restoring the mitochondria damaged by the toxicity of Aβ.

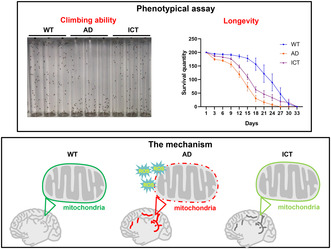

## 1 INTRODUCTION

Alzheimer's disease (AD) is the most prevalent form of senile dementia, mainly characterized by the presence of β‐amyloid (Aβ) accumulation and deposition.^1^, ^2^ And the latest and mainstream amyloid cascade hypothesis indicates that the main pathological changes in AD, including oxidative stress, energy metabolism damage, synaptic damage, glial cell activation, inflammation, and Tau protein hyperphosphorylation, are all attributed to the accumulation of soluble Aβ.^3^, ^4^ Hence, the development of therapeutic drugs for the accumulation of Aβ could still be the mainstream for dealing with AD.

*Drosophila* has been extensively used to build AD models due to its small size, ease of reproduction, and ease of genetic manipulation.^5^, ^6^, ^7^ *Drosophila* AD models were almost generated by expression of human Aβ, APP, presenilin, and BACE.^5^, ^6^, ^7^, ^8^, ^9^, ^10^, ^11^ These AD models exhibited AD‐like pathological and behavioral changes.^5^, ^6^, ^7^, ^8^, ^9^, ^10^, ^11^, ^12^, ^13^, ^14^ Arctic mutant Aβ_42_ (E22G) is an important mutation of Aβ_42_ in familial Alzheimer's disease (FAD).^15^ Expression of Aβ_arc_ (arctic mutant Aβ_42_) was more neurotoxic than expression of Aβ_40_ and Aβ_42_ in *Drosophila* ^13^, ^16^ consistent with the aggregative abilities of these peptides found in vitro.^17^ The declined flight ability and the shortened lifespan also occurred first in Aβ_arc_ *Drosophila*, followed by Aβ_42_ and Aβ_40_ *Drosophila*.^13^ That is to say, Aβ_arc_ *Drosophila* is an excellent *Drosophila* AD model to explore drugs for AD treatment.

Icaritin is a prenylated flavonoid, extracted from *Herba epimedii*, commonly used in Chinese traditional medicine.^18^ Nowadays, numerous pre‐clinical and clinical studies have investigated that Icaritin can fight for a variety of cancers, such as hepatocellular carcinoma (HCC),^19^ breast cancer,^20^ glioblastoma,^21^ and leukemia.^22^ And Icaritin displayed that only 6.9% of adverse reactions and no severe (grade 3–4) event occurred in clinical trials, which implied that Icaritin is convincingly safe.^23^ More importantly, the Icaritin soft capsule has been approved by the China National Medical Products Administration (NMPA) for the treatment of advanced HCC (https://www.nmpa.gov.cn/datasearch/search‐result.html).

Studies on the mechanism have shown that Icaritin exerts excellent antioxidant stress and anti‐inflammatory properties.^24^, ^25^ Oxidative stress and neuroinflammation have always been considered important pathological features of AD.^1^, ^2^ However, the study of Icaritin in AD has still been in a relatively blank state so far. Excitedly, liquid chromatograph mass spectrometer (LC–MS) results showed that Icaritin can pass through the blood–brain barrier in rat brain tissue after intragastric administration.^26^ Therefore, it is necessary to comprehensively clarify the neuroprotective effect of Icaritin on AD in vivo.

In this study, we first found that Icaritin greatly diminishes the toxicity of Aβ_arc_ in Aβ_arc_ *Drosophila*. In detail, Icaritin almost completely rescued the damaged climbing ability, flight ability, and longevity caused by Aβ_arc_ accumulation. We also found that Icaritin significantly reduces the oxidative stress caused by reactive oxygen species (ROS) and hydrogen peroxide and greatly improves the impaired energy metabolism.

Transcriptome analysis showed that Icaritin dramatically restores the expression of a large number of disorderly expressed genes in Aβ_arc_ *Drosophila*. Especially, many key genes, directly involved in oxidative stress and energy metabolism, were destructively destroyed by Aβ_arc_ toxicity and perfectly repaired by Icaritin treatment. In antioxidant stress, we found that Icaritin directly recovers the expression of peroxidase, superoxide dismutase, and glutathione s‐transferase (GST) genes damaged by Aβ_arc_ toxicity. In anti‐energy metabolism damage, we found that Icaritin recovers the expression of many key genes directly involved in glycolysis, the tricarboxylic acid (TCA) cycle, oxidative phosphorylation, and fatty acid β‐oxidation.

Numerous previous studies have shown that the integrity of mitochondrial structure and function is the key to maintaining the body's redox homeostasis and adequate energy metabolism.^27^, ^28^, ^29^ Excitedly, we found that Icaritin almost entirely recovers the integrity of mitochondrial structure and function destroyed by Aβ_arc_ toxicity. This indicated that mitochondrial repair may be a key process for Icaritin to rescue Aβ_arc_ *Drosophila*.

In summary, our study implied that Icaritin is a highly competitive potential natural small‐molecule drug for anti‐AD, which is realized at least partly through restoring the mitochondria/oxidative stress/energy metabolism axis.

## 2 MATERIALS AND METHODS

### 2.1 *Drosophila* stocks and genetics

*Drosophila* stocks were cultured under standard conditions. Briefly, after pupation, the adult flies were cultured on standard medium: ddH_2_O 0.65 L/L, baker's yeast 15 g/L, corn flour 38.85 g/L, agar 5.6 g/L, sucrose 15.81 g/L, glucose 31.6 g/L, and methyl p‐hydroxybenzoate 0.75 g/L (soluble in alcohol 7.5 mL) and entrained into a 12 h/12 h light/dark cycle at 25°C at a relative humidity of 50%–70%. The wild‐type W^1118^ (Bloomington stock, #5905) was used as a control and purchased from the *Drosophila* Bloomington Stock Center (University of Indiana, Bloomington, IN). The upstream activating sequence (UAS) transgenic lines (UAS‐Aβ_arc_) used for expressing the arctic mutant Aβ_42_ were generous gift from Dr. Fu‐De Huang (Institute of Neuroscience and State Key Laboratory of Neuroscience, Shanghai, China),^13^ in which the recombinant Aβ_arc_ DNAs were fused to a secretion signal of the *Drosophila* necrotic gene. The P[Gal4]A307^30^ was used to drive the expression of Aβ_arc_ in the GF system and other components of the nervous system.

### 2.2 Drug treatment

Icaritin (Yuanye, Shanghai, China, #118525‐40‐9) was dissolved in DMSO and then diluted to the final concentrations with ddH_2_O (dilution ratio: 1:1000) in this study. Eighty microliters of Icaritin solution with the final concentrations (0 μM, 10 μM, 30 μM, 50 μM) were daily added to the culture vial (with sufficient food) containing 20 newly eclosed subject flies (1–2‐day‐old) until 25 days. The administration method was completely based on a previous study.^31^ The flies were transferred to fresh food every 7 days.

### 2.3 Negative geotaxis assay

The climbing assay was performed according to the negative geotaxis climbing assay or automatic rapid iterative negative geotaxis (aRING) assay, as previously described.^32^ Briefly, a vial of flies (*n* ≥ 30, 25 days after eclosion) was tapped down to the bottom of the transparent testing vials with a diameter of 2.1 cm and a height of 19.0 cm. The flies were allowed to climb upwards on the walls of the tubes due to negative geotaxis. A digital video recorder mounted on a tripod 50 cm in front of the vial was used to record the climbing process of the flies. Flies were appraised in 3–5 consecutive trials separated by 60‐s intervals. The height of each fly in each vial at 40 s was measured by the software “RflyDetection” to evaluate the fly's climbing ability. All behavioral recording was done at 25°C.

### 2.4 Flight assay

The flight assay was performed as previously study described.^13^ Briefly, a single fly was tapped down into a glass cylinder with an inner diameter of 10 cm and a length of 39 cm, which was divided into 13 zones of 3 cm each. The zone in which the fly landed was recorded and used to estimate the landing height. Thirty flies were used in each group.

### 2.5 Longevity measurement

The longevity assay was evaluated as previously described.^13^ In summary, 200 flies from each group were equally separated into 10 vials containing standard fly food and cultured at 29°C. After every 3 days, the flies were transferred into vials with fresh food, and the number of dead flies was counted at that time. Survival quantities were analyzed with the GraphPad Prism software.

### 2.6 ELISA quantification of Aβ_arc_

The flies (*n* ≥ 50, 25 days after eclosion) were homogenized thoroughly in cold RIPA buffer (Solarbio, #R0020) supplemented with cocktail protease inhibitor (bimake, #B14001) with tissue homogenizer (Next Advance) and incubated on ice for 30 min. Samples were centrifuged at 13800 g for 10 min at 4°C, and the supernatants were collected for the ELISA analysis. The determination of the level of Aβ_arc_ used the Aβ_42_ Human ELISA Kit (Invitrogen, #KHB3441), according to the manufacturer's instructions.

### 2.7 Determination of redox system

The flies (*n* ≥ 50, 25 days after eclosion) were homogenized thoroughly in cold RIPA buffer (Solarbio, #R0020) supplemented with cocktail protease inhibitor (bimake, #B14001) with tissue homogenizer (Next Advance) and incubated on ice for 30 min. Samples were centrifuged at 13800 g for 10 min at 4°C, and the supernatants were collected for the redox system analysis. The determination of the content of ROS, hydrogen peroxide, and MDA used the Tissue Reactive Oxygen Species Detection Kit (Bestbio, #BB‐470512), Hydrogen Peroxide Assay Kit (Beyotime, #S0038), and Lipid Peroxidation MDA Assay Kit (Beyotime, #S0131S), respectively, according to the manufacturer's instructions. The determination of the activity of SOD, catalase, GST, and Caspase‐3 used the Total Superoxide Dismutase Assay Kit with WST‐8 (Beyotime, #S0101S), Catalase Assay Kit (Beyotime, #S0051), GST Activity Assay Kit (Solarbio, #BC0355), and Caspase‐3 Activity Assay Kit (Beyotime, #C1116), respectively, according to the manufacturer's instructions.

### 2.8 Determination of energy system

The flies (*n* ≥ 50, 25 days after eclosion) were homogenized thoroughly in cold RIPA buffer (Solarbio, #R0020) supplemented with cocktail protease inhibitor (bimake, #B14001) with tissue homogenizer (Next Advance) and incubated on ice for 30 min. Samples were centrifuged at 13800 g for 10 min at 4°C, and the supernatants were collected for the energy system analysis. The content of ATP and lactate was determined by applying the ATP Assay Kit (Beyotime, #S0026) and Lactic Acid Assay Kit (Sigma‐Aldrich, MAK064), respectively, according to the manufacturer's instructions.

### 2.9 Immunostaining and imaging

The brains of flies were dissected in pre‐cooling PBS and fixed with 4% paraformaldehyde in PBS for 1 h, washed with PBS‐Triton X‐100 (0.3%) for 5 min and repeated five times, and then treated with formic acid (70% in water) for 45 min to reexpose the epitope. Preparations were then washed three times with PBS‐Triton X‐100 (0.3%) for 5 min, blocked with 5% normal goat serum (Solarbio, #SL038) in PBS‐Triton X‐100 (0.3%) at room temperature for at least 30 min. The samples were then incubated with primary antibodies (β‐amyloid, 1:100, Covance, #SIG‐39300; Cleaved Caspase‐3, 1:50, Cell Signaling Technology, #9664) overnight at 4°C, washed with PBS‐Triton X‐100 (0.3%) for 10 min and repeated five times, and then incubated with (ZSGB‐BIO, 1:100, #ZF‐0315) or 594‐conjugated secondary anti‐rabbit antibody (Cell Signaling Technology, 1:500, #8889) at room temperature for 2 h in the dark, and then confocal imaging. Imaged with a laser scanning confocal microscope (Olympus FV3000) at a step of 1 μm to acquire the projection of Z‐stack images. A standard image of Aβarc or Cleaved Caspase‐3 in the Aβ_arc_ fly was taken, and then the imaging parameters of it were used for imaging on all the rest flies.

### 2.10 Transcriptome analysis

The flies (*n* ≥ 50, 25 days after eclosion) were dissected in pre‐cooling PBS, and total RNA was extracted using RNAiso Plus (TaKaRa, China). After a quality check, the RNA was processed for RNA sequencing by Novogene Co., Ltd (Beijing, China). Differentially expressed genes (DEGs) with |log2 (fold change)| >0 and *p* values <0.05 were considered significant. GO pathway analysis and KEGG pathway analysis were performed with the NovoMagic system (https://magic.novogene.com).

### 2.11 Quantitative real‐time PCR (qRT‐PCR)

To measure the expression levels of genes in *Drosophila*, 20 flies, 25 days after eclosion, were homogenized thoroughly with 1 mL of TRI reagent (MRC, #TR118), and total RNAs were extracted according to the manufacturer's instructions. cDNA synthesis was performed using the PrimeScript™ RT Reagent Kit with the gDNA Eraser (Takara, #RR047A). And qRT‐PCR was performed by the Bio‐Rad CFX96 PCR System with MonAmp™ SYBR® Green qPCR Mix (Monad, #MQ10101S) according to the manufacturer's instructions. The expression level of each target gene was normalized to 18S. The primers used are listed in Table S1.

### 2.12 Electron microscopy

The thoraces of flies were dissected, fixed in 2.5% glutaraldehyde and 1% osmium tetroxide, and embedded in Epon resin according to standard procedures optimized for *Drosophila* tissue.^33^ Ultra‐thin sections were stained with uranyl acetate and lead citrate. HT7700 TEM (HITACHI, Japan) was used for observation. Broken mitochondria were quantified by manual counting.

### 2.13 Mitochondrial respiration assay

Mitochondrial respiration was measured at 37°C by using the Oxygraph‐2 K high‐resolution respirometry (Oxygraph‐2 K 10000‐1, Oroboros, AT). Respiration was assayed by homogenizing 10 flies on ice using a pestle in MiR05 respiration buffer (20 mM HEPES, 10 mM KH_2_PO_4_, 110 mM sucrose, 20 mM taurine, 60 mM K‐lactobionate, 0.5 mM EGTA, 3 mM MgCl_2_, and 1 g/L fatty acid‐free BSA). Next, various substrates, uncouplers, and inhibitors for mitochondrial respiratory chain complexes were used as follows: substrates including 2 M pyruvate, 0.8 M malate, 2 M glutamate, 1 M succinate, 0.5 M ADP + Mg^2+^, and 4 mM cytochrome C; uncouplers including 1 mM carbonyl cyanide m‐chlorophenyl hydrazine; and inhibitors including 1 mM rotenone and 5 mM antimycin A. Complex І respiration was measured in MiR05 respiration buffer in the presence of pyruvate, malate, glutamate, and ADP + Mg^2+^. Complex II was assayed in respiration buffer supplemented with rotenone and succinate. Oxygen concentration and oxygen flux indicating Complex І and Complex II function were recorded using DatLab software (Oroboros Instruments) as previously described.^34^

### 2.14 Assessment of mitochondrial membrane potential

Mitochondria were extracted from 50 flies (25 days after eclosion) in each group using the Tissue Mitochondria Isolation Kit (Beyotime, #C3606). Mitochondrial membrane potential was evaluated by the Enhanced Mitochondrial Membrane Potential Assay Kit with JC‐1 (Beyotime, #C2003S) according to the manufacturer's instructions.

### 2.15 Statistical analysis

GraphPad Prism software was used for data analysis. A one‐way ANOVA followed by Tukey's post hoc test was used for statistical analysis when all groups were compared among them, or a two‐tailed unpaired Student's *t*‐test was used for statistical analysis when two groups were compared. Significance was considered at *p ≤ 0.05, **p ≤ 0.01, ***p ≤ 0.005, or ****p ≤ 0.001 throughout the study. The data were represented as mean ± SD. All experiments were run at least in triplicate and annotated as “*n*” in the corresponding figure legends.

## 3 RESULTS

### 3.1 Icaritin rescues Aβ_arc_ *Drosophila*

**FIGURE 1:**
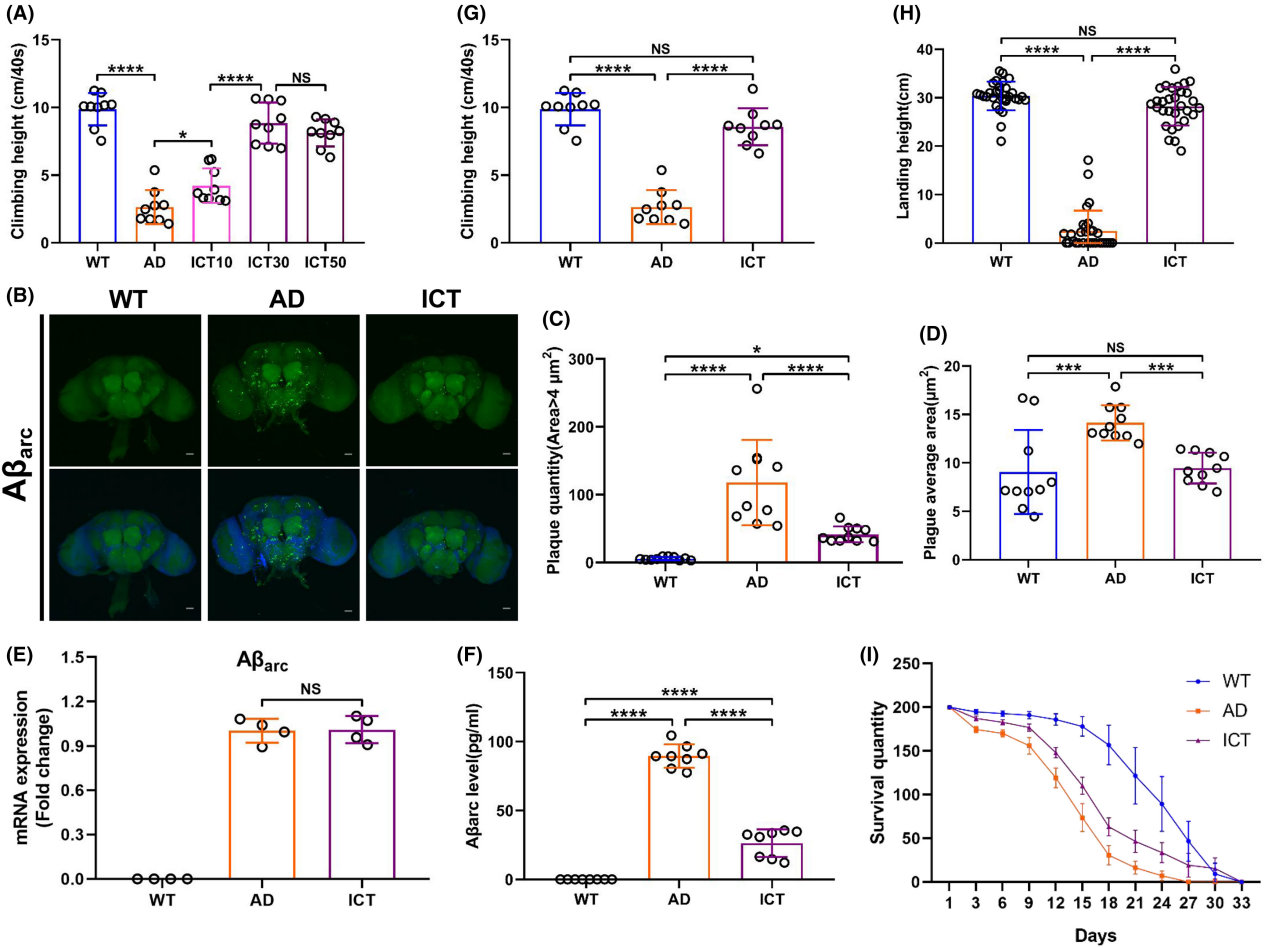
Icaritin rescues the climbing ability, flight ability, and longevity of Aβ_arc_ *Drosophila*. (A) Determination of the climbing ability in each group (*n* = 30 in each vial) and the concentrations of Icaritin used are 0, 10, 30, and 50 μM. (B) Immunostaining of Aβ_arc_ (whole brain; *n* = 10). (C, D) Quantitative analysis of (B). (E) Determination of the mRNA levels of Aβ_arc_. (F) Determination of soluble Aβ_arc_ content. (G–I) Determination of climbing ability (*n* = 30 in each vial) (G), flight ability (*n* = 30) (H), and longevity analysis (I). WT: wild‐type group; AD, Aβ_arc_ expression group; ICT, Aβ_arc_ expression plus Icaritin 30 μM treatment group. Error bars represent the SD of at least eight independent experiments. NS represents not significant. Scale bars, 50 μm. *p ≤ 0.05, **p ≤ 0.01, ***p ≤ 0.005, and ****p ≤ 0.001 use a one‐way ANOVA followed by Tukey's post hoc test and an unpaired two‐tailed Student's *t*‐test.

The Aβ_arc_ *Drosophila* was used to study the neuroprotective effects of Icaritin on AD. First, we investigated that the beneficial effect of Icaritin on climbing ability is dose dependent (Figure 1A). The optimal concentration of Icaritin was determined to be 30 μM (Figure 1A). In the subsequent study, three groups were generated. Wild‐type group (WT): A307 > W^1118^, Aβ_arc_ expression group (AD): A307 > Aβ_arc_, and Aβ_arc_ expression plus Icaritin treatment group (ICT): A307 > Aβ_arc_ + ICT 30 μM. We found that Icaritin successfully attenuates the accumulation and deposition of Aβ_arc_ in the brain of Aβ_arc_ *Drosophila* by immunostaining (Figure 1B–D). We also found that Icaritin significantly reduces soluble Aβ_arc_ in Aβ_arc_ *Drosophila* by ELISA (Figure 1F). The result of qRT‐pCR showed that the mRNA levels of Aβ_arc_ did not change between the AD and ICT groups (Figure 1E). Behavioral analysis revealed that Icaritin almost completely rescues the climbing ability, flight ability, and longevity declined by Aβ_arc_ toxicity (Figure 1G–I). Moreover, we noticed that the survival quantity of the ICT group was higher than that of the WT group on Day 30 (Figure 1I). These results indicated that Icaritin successfully rescues Aβ_arc_ *Drosophila*.

### 3.2 Icaritin restores the unbalanced redox homeostasis in Aβ_arc_ *Drosophila*

**FIGURE 2:**
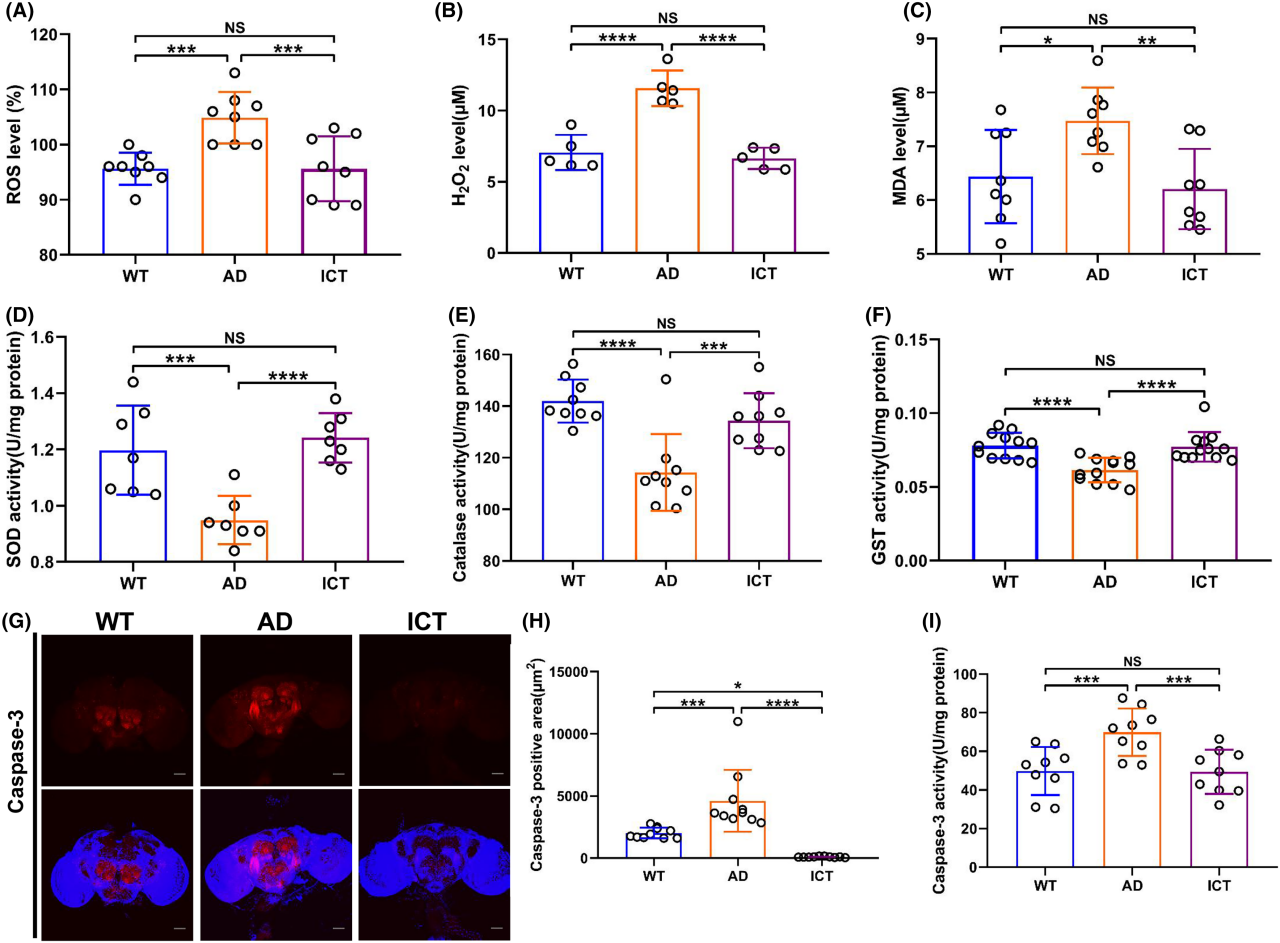
Icaritin restores the unbalanced redox homeostasis of Aβ_arc_ *Drosophila*. (A, B) Detect the content of ROS (A) and hydrogen peroxide (B). (C) Analyzing membrane lipid peroxidation levels by measuring MDA. (D–F) Analyzing the antioxidant enzyme system by confirming the activity of SOD (D), catalase (E), and GST (F). (G) Immunostaining of active Caspase‐3 (whole brain; *n* = 10). (H) Quantitative analysis of (G). (I) Determination the activity of Caspase‐3. WT, wild‐type group; AD, Aβ_arc_ expression group; ICT, Aβ_arc_ expression plus Icaritin 30 μM treatment group. Error bars represent the SD of at least five independent experiments. NS represents not significant. Scale bars, 50 μm. *p ≤ 0.05, **p ≤ 0.01, ***p ≤ 0.005, and ****p ≤ 0.001 use a one‐way ANOVA followed by Tukey's post hoc test.

The unbalanced redox homeostasis is an important feature of AD. First, we found that Icaritin significantly reduces the increased ROS and hydrogen peroxide in Aβ_arc_ *Drosophila* (Figure 2A,B). Second, Icaritin effectively reduced the increased MDA in Aβ_arc_ *Drosophila* and attenuated the level of lipid peroxidation (Figure 2C). Third, Icaritin greatly restored the activity of SOD, catalase, and GST, which were damaged by Aβ_arc_ toxicity in Aβ_arc_ *Drosophila* (Figure 2D–F). Additionally, we found that Icaritin dramatically attenuates apoptosis, which is induced by Aβ_arc_ toxicity in Aβ_arc_ *Drosophila*, by immunostaining the brain with active Caspase‐3 (Figure 2G,H). This result was further supported by the determination of Caspase‐3 activity by ELISA (Figure 2I). Interestingly, the immunostaining result also showed that the active Caspase‐3 positive signal in the ICT group is much weaker than that in the WT group (Figure 2G). These results suggested that Icaritin restores the unbalanced redox homeostasis and repairs the related damage in Aβ_arc_ *Drosophila*.

### 3.3 Icaritin repairs the damaged energy metabolism in Aβ_arc_ *Drosophila*

**FIGURE 3:**
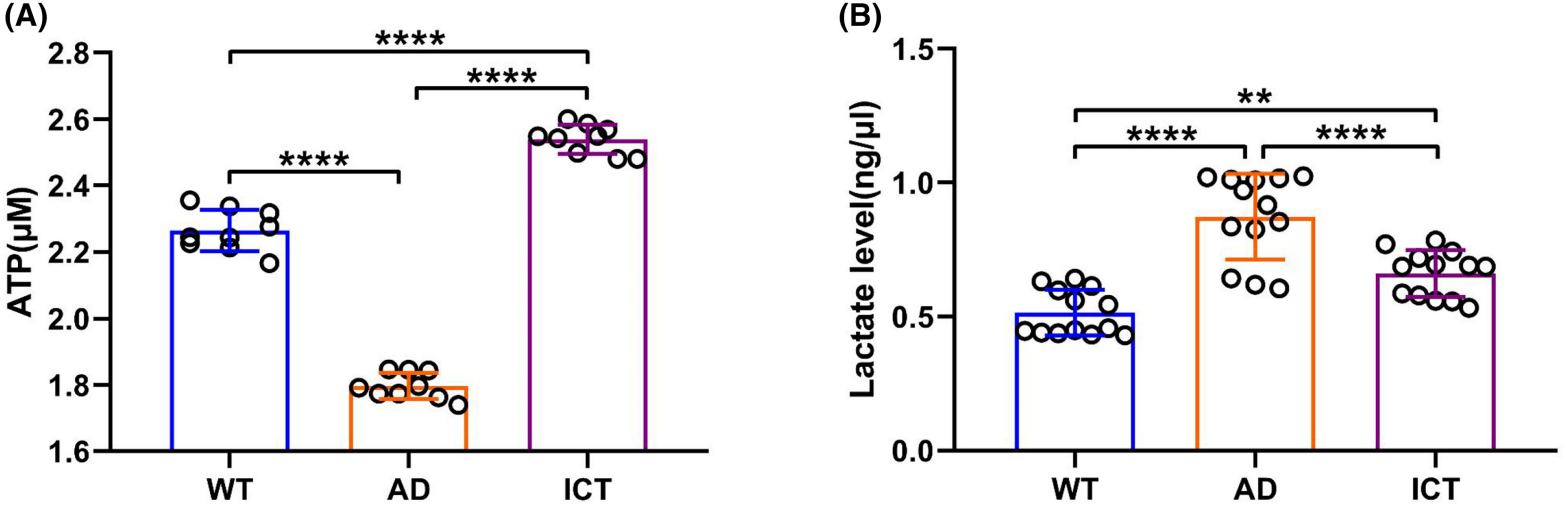
Icaritin repairs the energy metabolism of Aβ_arc_ *Drosophila*. (A) Analyzing brain energy supply by measuring the content of ATP. (B) Determination of the content of lactate. AD, Aβ_arc_ expression group; ICT, Aβ_arc_ expression plus Icaritin 30 μM treatment group; WT, wild‐type group. Error bars represent the SD of at least nine independent experiments. *p ≤ 0.05, **p ≤ 0.01, ***p ≤ 0.005, and ****p ≤ 0.001 use a one‐way ANOVA followed by Tukey's post hoc test.

The damaged energy metabolism is another important feature of AD. First, we found that Icaritin greatly restores ATP production damaged by Aβ_arc_ toxicity in Aβ_arc_ *Drosophila* (Figure 3A). Second, Icaritin treatment significantly decreased the lactate level in Aβ_arc_ *Drosophila* (Figure 3B). Interestingly, we also found that the content of ATP in the ICT group is more than that in the WT group (Figure 3A). These results suggested that Icaritin dramatically restores the energy supply of the nervous system, which is damaged by Aβ_arc_ toxicity.

### 3.4 Transcriptome analysis

**FIGURE 4:**
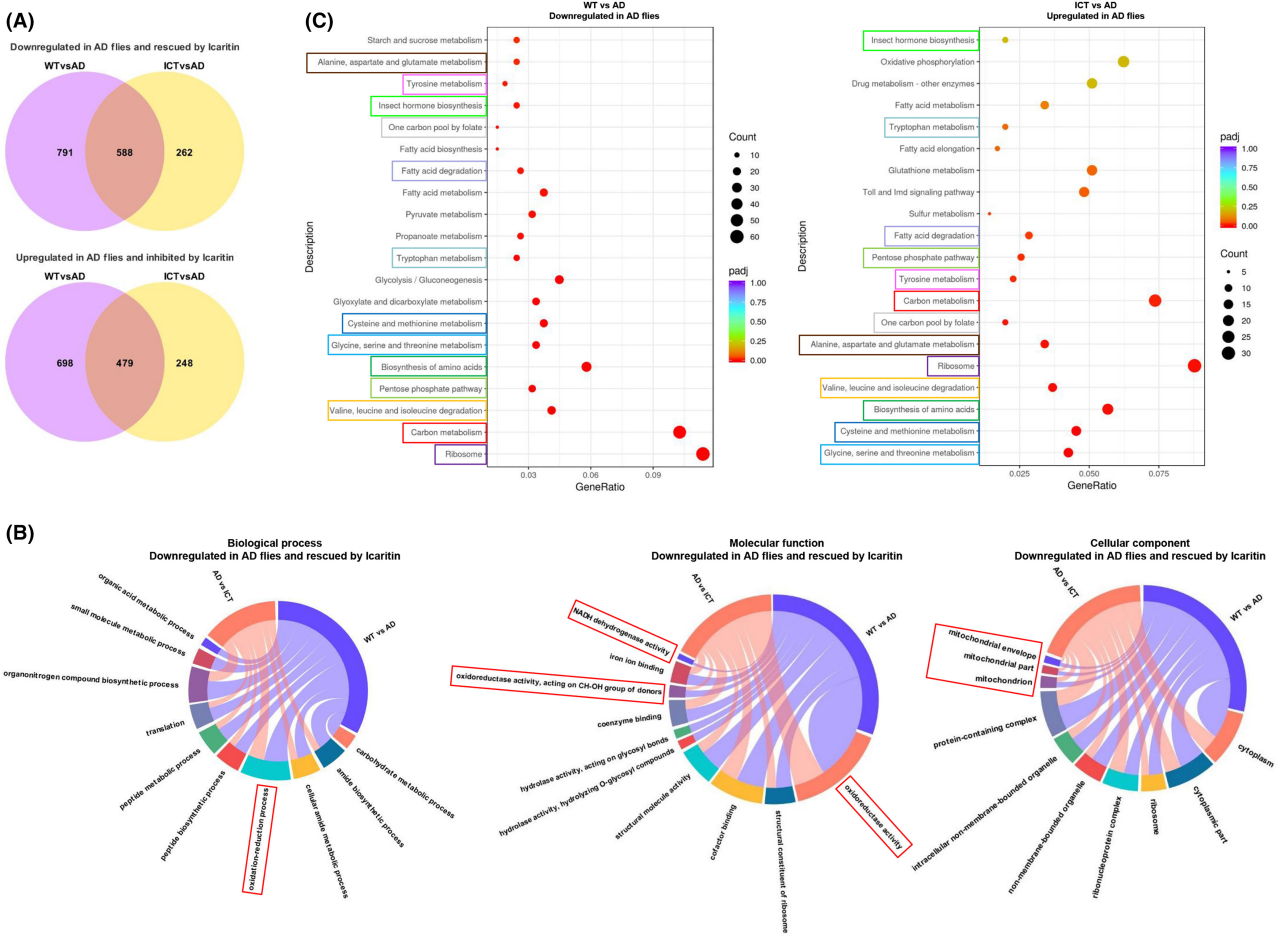
Transcriptome analysis. (A) A Venn diagram analyses the count of differentially expressed genes. (B) GO pathway analysis confirms the biological significance of differentially expressed genes. (C) KEGG pathway analysis confirms the most significantly affected pathways, and boxes of the same color represent pathways damaged by Aβarc toxicity and rescued by Icaritin treatment. AD, Aβ_arc_ expression group; ICT, Aβ_arc_ expression plus Icaritin 30 μM treatment group; WT, wild‐type group. *n* = 3.

To elucidate the molecular events occurring during Aβ_arc_ expression and Icaritin treatment, transcriptome analysis was performed. Expression of 1379 genes was downregulated and expression of 1177 genes was upregulated in Aβ_arc_ *Drosophila* (Tables S2 and S3), of which expression of 588 genes was upregulated and expression of 479 genes was downregulated by Icaritin, respectively (Figure 4A, Tables S4 and S5). To better understand the biological significance of the above genes, gene ontology (GO) pathway analysis was performed to obtain the biological information comments from the aspects of biological process, cellular component, and molecular function (Figure 4B). Impressively, the oxidation–reduction process was rescued by Icaritin treatment from the aspect of biological processes (Figure 4B). Oxidoreductase activity and NADH dehydrogenase activity were rescued by Icaritin treatment from the aspect of molecular function (Figure 4B). Mitochondria were rescued by Icaritin treatment from the aspect of cellular component (Figure 4B). A Kyoto Encyclopedia of Genes and Genomes (KEGG) pathway analysis was conducted, and the top 20 pathways of different groups were listed (Figure 4C). Most of the pathways were rescued by Icaritin treatment (Figure 4C). Importantly, carbon metabolism was the most severely damaged and best rescued (Figure 4C). These results indicated that Icaritin rescues Aβ_arc_ *Drosophila* by systematically repairing multiple important metabolic processes damaged by Aβ_arc_ toxicity, especially oxidative stress and energy metabolism related to mitochondria.

### 3.5 The key genes involved in antioxidant stress are repaired

**FIGURE 5:**
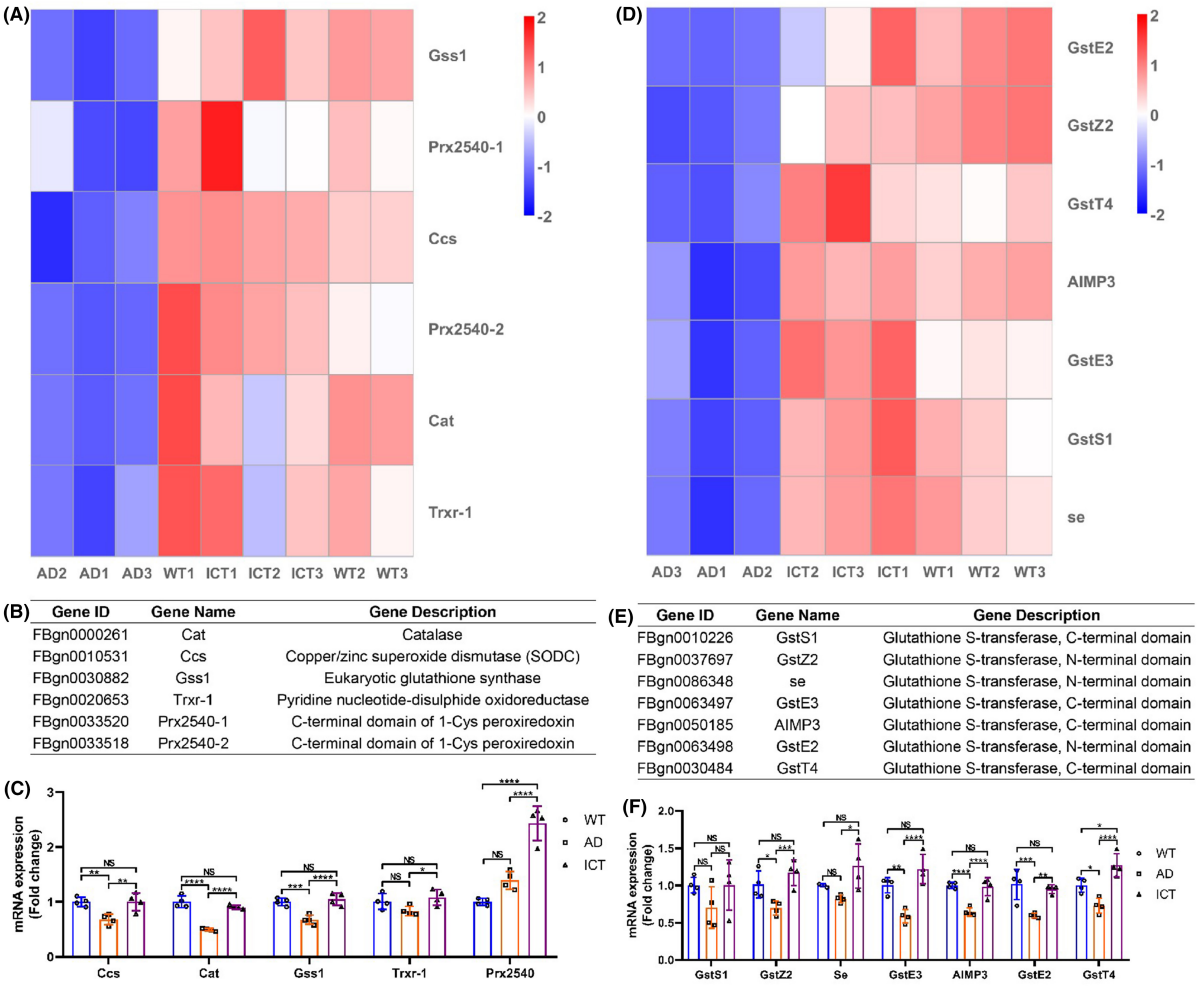
Icaritin restores the expression of genes in redox homeostasis. (A–C) Heatmap analysis of the transcriptome data of the expression of enzymatic genes directly involved in antioxidant stress (A) gene description (B) and qRT‐PCR verification (C). (D, F) Heatmap analysis of the transcriptome data of the expression of GST genes directly involved in internal detoxification (D), gene description (E), and qRT‐PCR verification (F). WT1, WT2, and WT3 represent three independent wild‐type groups; AD1, AD2, and AD3 represent three independent Aβ_arc_ expression groups; and ICT1, ICT2, and ICT3 represent three independent Aβ_arc_ expression plus Icaritin 30 μM treatment groups. Error bars represent the SD of four independent experiments. NS represents not significant. *p ≤ 0.05, **p ≤ 0.01, ***p ≤ 0.005, and ****p ≤ 0.001 use a one‐way ANOVA followed by Tukey's post hoc test.

Combined with the transcriptome data, we further analyzed the genes directly related to the maintenance of redox homeostasis. The heatmap of transcriptome data showed that Aβ_arc_ toxicity downregulates the expression of Cat, Ccs, Gss1, Trxr‐1, and Prx2540, which is upregulated by Icaritin treatment (Figure 5A). The gene descriptions of Cat, Ccs, Gss1, Trxr‐1, and Prx2540 are shown in Figure 5B. The qRT‐PCR results verified that the expression of Cat, Ccs, and Gss1 is consistent with that of the transcriptome data (Figure 5C). The expression of Trxr‐1 and Prx2540 was unchanged in Aβ_arc_ *Drosophila*, which was inconsistent with the results of transcriptome data, while Icaritin activated the expression of Trxr‐1 and Prx2540 (Figure 5C). Additionally, the transcriptome data showed that Aβ_arc_ toxicity downregulates the expression of CG31028 and Cd, which is not restored by Icaritin treatment (Figure S1A). The gene descriptions of CG31028 and Cd are shown in Figure S1B. But the qRT‐PCR results confirmed that the expression of CG31028 is downregulated under Aβ_arc_ expression and recovered by Icaritin treatment, while the expression of Cd is unchanged by Aβ_arc_ expression and Icaritin treatment (Figure S1C).

The heatmap of transcriptome data also showed that Aβ_arc_ toxicity downregulates the expression of GstS1, GstZ2, Se, GstE3, AIMP3, GstE2, and GstT4, which is upregulated by Icaritin treatment (Figure 5D). The gene descriptions of GstS1, GstZ2, Se, GstE3, AIMP3, GstE2, and GstT4 are shown in Figure 5E. The qRT‐PCR results verified that the expression of GstZ2, GstE3, AIMP3, GstE2, and GstT4 is consistent with that of the transcriptome data (Figure 5F). The expression of Se was unchanged in Aβ_arc_ *Drosophila*, which was inconsistent with the results of transcriptome data, while Icaritin greatly activated the expression of Se (Figure 5F). The expression of GstS1 was unchanged under Aβ_arc_ expression and Icaritin treatment, which was inconsistent with the results of transcriptome data (Figure 5F). Additionally, the transcriptome data showed that Aβ_arc_ toxicity downregulates the expression of GstE8, GstE14, eEF1 gamma, GstE11, and Gfzf in Aβ_arc_ *Drosophila*, which is not restored by Icaritin treatment (Figure S1D). The gene descriptions of GstE8, GstE14, eEF1 gamma, GstE11, and Gfzf are shown in Figure S1E. But the qRT‐PCR results verified that the expression of GstE8, eEF1 gamma, GstE11, and Gfzf is downregulated under Aβarc expression and recovered by Icaritin treatment, while the expression of GstE14 is unchanged by Aβarc expression and Icaritin treatment (Figure S1F).

These results manifested that Icaritin restores the expression of enzymes, which are directly involved in the antioxidant enzyme system and damaged by Aβ_arc_ toxicity, to rebalance the imbalance of redox homeostasis in Aβ_arc_ *Drosophila*.

### 3.6 The key genes involved in energy metabolism are repaired

Combined with the transcriptome data, we further analyzed the expression of the genes directly related to glycolysis, TCA cycle, oxidative phosphorylation, pentose phosphate pathway, and fatty acid β‐oxidation.

**FIGURE 6:**
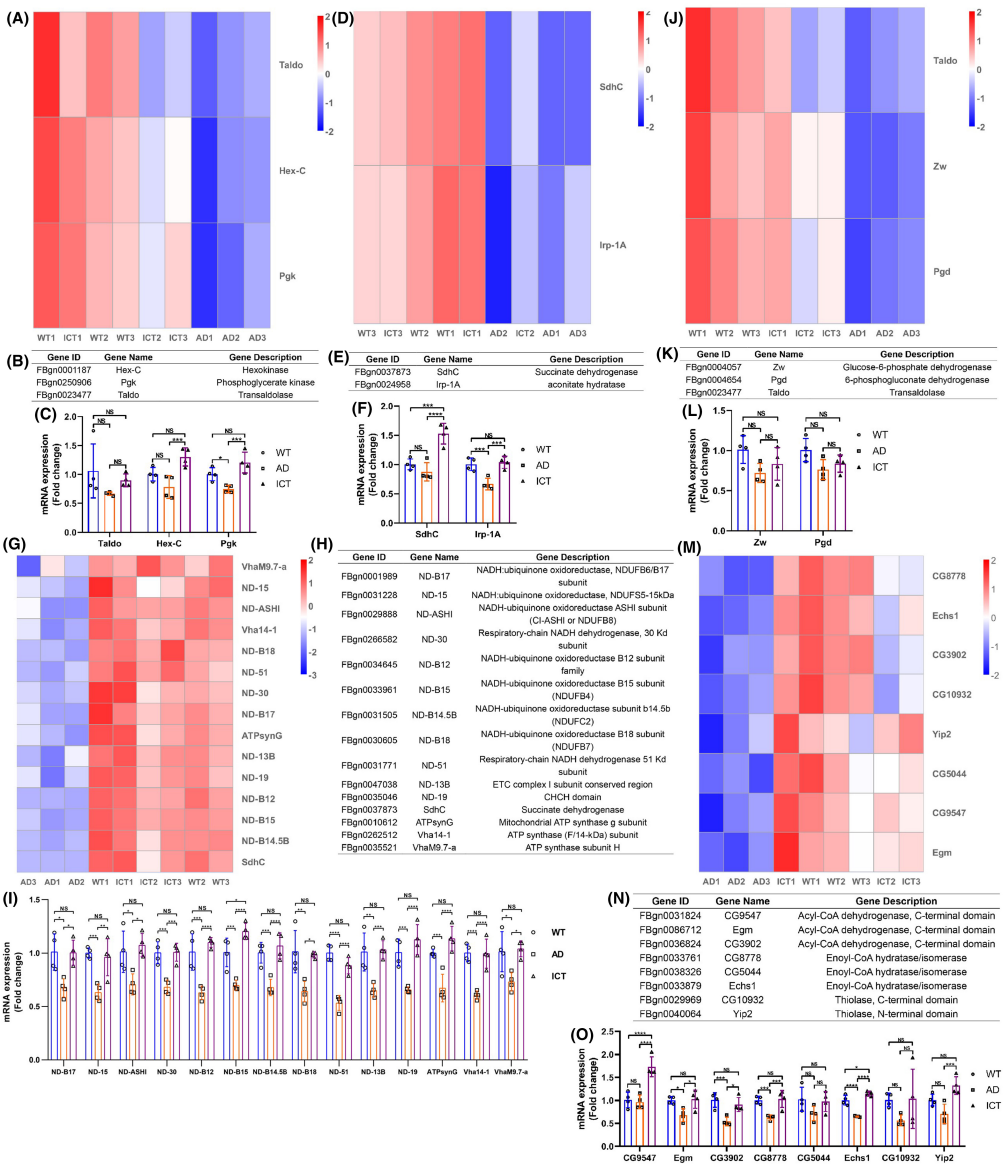
Icaritin restores the expression of genes in energy metabolism. (A–C) In glycolysis, heatmap analysis of the transcriptome data of the expression of enzymatic genes directly involved in glycolysis (A), gene description (B), and qRT‐PCR verification (C). (D–F) In the TCA cycle, heatmap analysis of the transcriptome data of the expression of enzymatic genes directly involved in TCA cycle (D), gene description (E), and qRT‐PCR verification (F). (G–I) In oxidative phosphorylation, heatmap analysis of the transcriptome data of the expression of genes directly encoding important components of Complex I and ATP synthetase (G), gene description (H), and qRT‐PCR verification (I). (J–L) In the pentose phosphate pathway, heatmap analysis of the transcriptome data of the expression of enzymatic genes directly involved in pentose phosphate pathway (J), gene description (K), and qRT‐PCR verification (L). (M–O) In fatty acid β‐oxidation, heatmap analysis of the transcriptome data of the expression of enzymatic genes directly involved in fatty acid β‐oxidation (M), gene description (N), and qRT‐PCR verification (O). WT1, WT2, and WT3 represent three independent wild‐type groups; AD1, AD2, and AD3 represent three independent Aβ_arc_ expression groups; ICT1, ICT2, and ICT3 represent three independent Aβ_arc_ expression plus Icaritin 30 μM treatment groups. Error bars represent the SD of four independent experiments. NS represents not significant. *p ≤ 0.05, **p ≤ 0.01, ***p ≤ 0.005, and ****p ≤ 0.001 use a one‐way ANOVA followed by Tukey's post hoc test.

In glycolysis, the heatmap of transcriptome data showed that Aβ_arc_ toxicity downregulates the expression of Hex‐C, Pgk, and Taldo in Aβ_arc_ *Drosophila*, which is upregulated by Icaritin treatment (Figure 6A). The gene descriptions of Hex‐C, Pgk, and Taldo are shown in Figure 6B. But the qRT‐PCR results verified that only the expression of Pgk is consistent with that of the transcriptome data (Figure 6C). The expression of Hex‐C was unchanged under Aβ_arc_ expression, while Icaritin activated the expression of Hex‐C (Figure 6C). The expression of Taldo remained unchanged under Aβ_arc_ expression and Icaritin treatment (Figure 6C). The transcriptome data also showed that Aβ_arc_ toxicity downregulates the expression of Pgi and Pfk in Aβ_arc_ *Drosophila*, which is not restored by Icaritin treatment (Figure S2A). The gene descriptions of Pgi and Pfk are shown in Figure S2B. But the qRT‐PCR results verified that the expression of Pgi is downregulated under Aβ_arc_ expression and restored by Icaritin treatment, while the expression of Pfk is unchanged under Aβ_arc_ expression, and Icaritin activates the expression of Pfk (Figure S2C). The above genes are all coding genes for important enzymes directly involved in the glycolytic pathway. These results showed that Icaritin repairs glycolysis pathway damaged by Aβ_arc_ toxicity.

In the TCA cycle, the heatmap of transcriptome data showed that Aβ_arc_ toxicity downregulates the expression of SdhC and Irp‐1A in Aβ_arc_ *Drosophila*, which is upregulated by Icaritin treatment (Figure 6D). The gene descriptions of SdhC and Irp‐1A are shown in Figure 6E. But the qRT‐PCR results verified that only the expression of Irp‐1A is consistent with that of the transcriptome data (Figure 6F). The expression of SdhC was unchanged under Aβ_arc_ expression, while Icaritin activated the expression of SdhC (Figure 6F). The transcriptome data also showed that Aβ_arc_ toxicity downregulates the expression of AcCoAS and ATPCL in Aβ_arc_ *Drosophila*, which is not restored by Icaritin treatment (Figure S2D). The gene descriptions of AcCoAS and ATPCL are shown in Figure S2E. But the qRT‐PCR results verified that the expression of AcCoAS is downregulated under Aβ_arc_ expression and restored by Icaritin treatment, while the expression of ATPCL remains unchanged under Aβ_arc_ expression, and Icaritin treatment (Figure S2F). The above genes are all coding genes for important enzymes directly involved in the TCA cycle pathway. These results showed that Icaritin repairs the TCA cycle pathway damaged by Aβ_arc_ toxicity.

In oxidative phosphorylation, the heatmap of transcriptome data showed that Aβ_arc_ toxicity downregulates the expression of 15 genes in Aβ_arc_ *Drosophila*, which is upregulated by Icaritin treatment (Figure 6G). The gene description of the 15 genes showed that all the genes are important components of Complex I and ATP synthetase (Figure 6H). The qRT‐PCR results verified that the expression of the 14 genes is consistent with that of the transcriptome data (Figure 6I), while the expression of SdhC is unchanged under Aβ_arc_ expression, and Icaritin activates the expression of SdhC (Figure 6F). The transcriptome data also showed that Aβ_arc_ toxicity downregulates the expression of other 9 genes in Aβ_arc_ *Drosophila*, which is not restored by Icaritin treatment (Figure S2G). The gene description of the 9 genes showed that all the genes are important components of Complex I and ATP synthetase (Figure S2H). The qRT‐PCR results verified that the expression of 8 genes is downregulated under Aβarc expression and restored by Icaritin treatment, while the expression of VhaPPA1‐1 remains unchanged under Aβ_arc_ expression, and Icaritin treatment activates the expression of SdhC (Figure S2I). The above genes are all coding genes for important components of the enzymes directly involved in the oxidative phosphorylation pathway. These results showed that Icaritin repairs the oxidative phosphorylation pathway damaged by Aβ_arc_ toxicity.

In pentose phosphate pathway, the heatmap of transcriptome data showed that Aβ_arc_ toxicity downregulates the expression of Zw, Pgd, and Taldo in Aβ_arc_ *Drosophila*, which is upregulated by Icaritin treatment (Figure 6J). The gene description of Zw, Pgd, and Taldo was showed in Figure 6K. However, the qRT‐PCR results verified that the expression of Zw, Pgd, and Taldo remains unchanged under Aβ_arc_ expression and Icaritin treatment (Figure 6C, L). The above genes are all coding genes for the enzymes directly involved in the pentose phosphate pathway. These results showed that pentose phosphate pathway may remain stable under Aβ_arc_ expression and Icaritin treatment.

In fatty acid β‐oxidation, the heatmap of transcriptome data showed that Aβ_arc_ toxicity downregulates the expression of eight genes in Aβ_arc_ *Drosophila*, which is upregulated by Icaritin treatment (Figure 6M). The gene descriptions of the eight genes are shown in Figure 6N. The qRT‐PCR results verified that the expression of Egm, Echs1, CG3902, and CG8778 is consistent with that of the transcriptome data (Figure 6O). The expression of CG9547 and Yip2 was unchanged under Aβ_arc_ expression, while Icaritin activated the expression of CG9547 and Yip2 (Figure 6O). The expression of CG5044 and CG10932 remained unchanged under Aβ_arc_ expression and Icaritin treatment (Figure 6O). The transcriptome data also showed that Aβ_arc_ toxicity downregulates the expression of other eight genes in Aβ_arc_ *Drosophila*, which is not restored by Icaritin treatment (Figure S2J). The gene descriptions of the eight genes are shown in Figure S2K. The qRT‐PCR results verified that the expression of ATPCL remains unchanged under Aβ_arc_ expression and Icaritin treatment (Figure S2F), and the expression of CG4594 was unchanged under Aβ_arc_ expression while Icaritin activates the expression of CG4594 (Figure S2L). The expression of the other six genes was downregulated under Aβarc expression and restored by Icaritin treatment (Figure S2L). The above genes are all coding genes for the enzymes directly involved in the fatty acid β‐oxidation pathway. These results showed that Icaritin repairs the fatty acid β‐oxidation pathway damaged by Aβ_arc_ toxicity.

These results indicated that Aβ_arc_ toxicity almost damages all the energy metabolism pathways, and Icaritin can systematically repairs the entire process of ATP production damaged in Aβ_arc_ *Drosophila*.

### 3.7 Icaritin restores the integrity of mitochondrial structure and function

**FIGURE 7:**
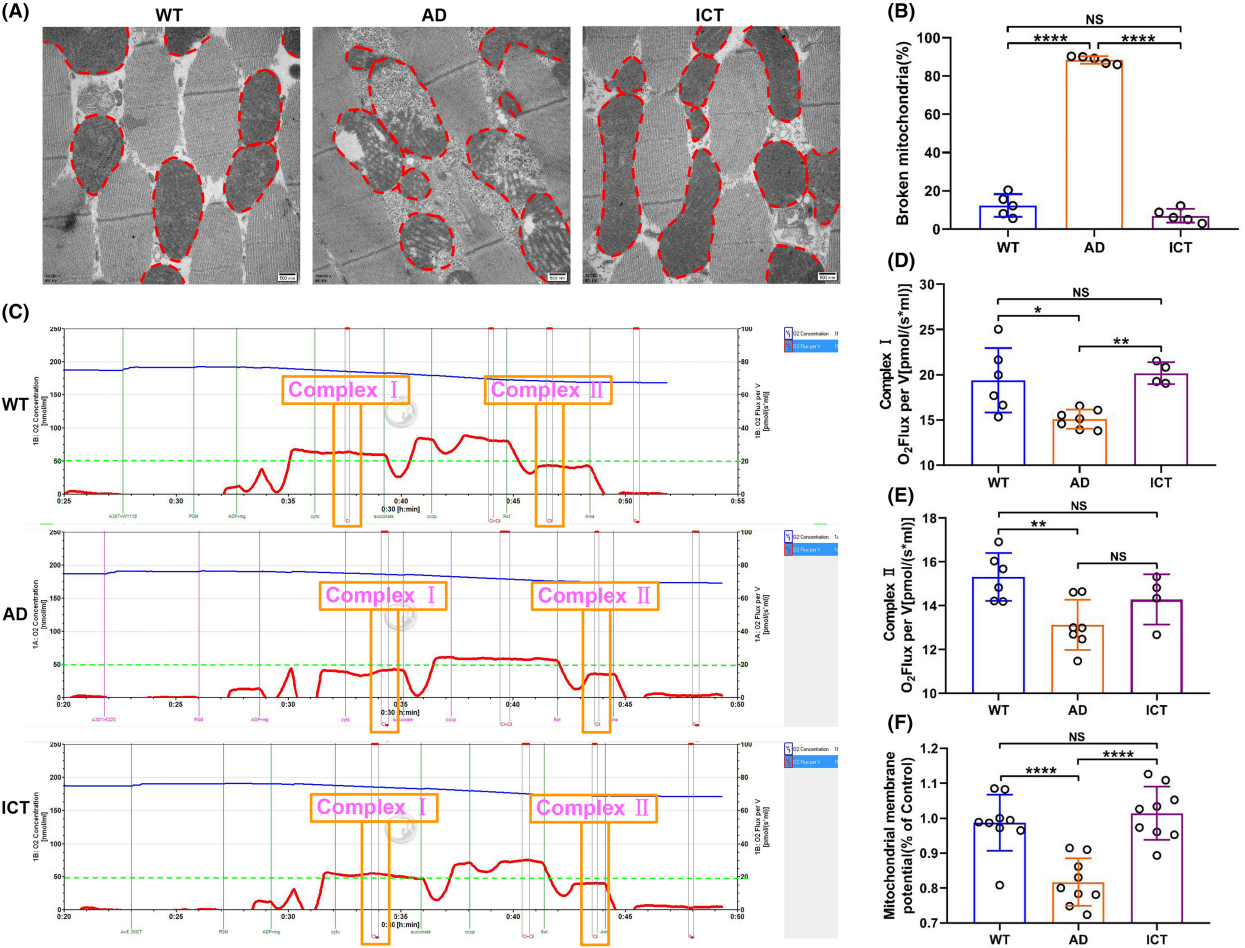
Icaritin repairs the damaged mitochondria in Aβ_arc_ *Drosophila*. (A, B) The red dashed line represents mitochondria in each group (A), quantitative analysis of structurally intact mitochondria (B). (C–E) mitochondrial respiration in each group (C), Complex І respiration (D), and Complex II respiration (E) are analyzed according to the mitochondrial respiration assay. (F) Determination of the mitochondrial membrane potential. AD, Aβ_arc_ expression group; ICT, Aβ_arc_ expression plus Icaritin 30 μM treatment group; WT: wild‐type group. Error bars represent the SD of at least four independent experiments. NS represents not significant. *p ≤ 0.05, **p ≤ 0.01, ***p ≤ 0.005, and ****p ≤ 0.001 use a one‐way ANOVA followed by Tukey's post hoc test. Scale bars, 500 nm.

Redox homeostasis and energy metabolism are closely related to mitochondria. Therefore, we detected the structural and functional integrity of mitochondria. First, we found that Icaritin restores the integrity of mitochondrial structure, which is damaged by Aβ_arc_ toxicity in Aβ_arc_ *Drosophila* (Figure 7A,B). Second, mitochondrial functional analysis showed that Aβ_arc_ toxicity disrupts the function of complex I and complex II, and the function of complex I is extremely restored by Icaritin treatment (Figure 7C–E). Moreover, mitochondrial membrane potential analysis also showed that Icaritin repairs the integrity of mitochondrial structures damaged in Aβ_arc_ *Drosophila* (Figure 7F). These results indicated that mitochondria may be the targeted organelle for Icaritin to rescue Aβ_arc_ *Drosophila*.

## 4 DISCUSSION

By expressing Aβ_arc_ in the GF system with a tissue‐specific promoter,^13^ we generated an excellent *Drosophila* AD model for investigating the neuroprotective effect of Icaritin on AD in vivo. We found that Aβ_arc_ exactly accumulates in the GF system, as previously studies described.^13^, ^16^ Climbing ability, flight ability, and longevity have always been used as classic phenotypes to evaluate the disease progression of AD *Drosophila*.^13^, ^35^ In this study, Icaritin almost completely rescued the climbing ability, flight ability, and longevity declined by Aβ_arc_ toxicity in *Drosophila*. The results indicated that Icaritin is a potential promising drug candidate for AD therapy. Moreover, we observed an interesting phenomenon: the survival quantity of ICT group was higher than that of the WT group on Day 30. This implies that the extension of lifespan by Icaritin may not be limited to AD. This might be related to the accumulation of beneficial effects caused by long‐term oral administration of Icaritin.

Oxidative stress is a bridge that connects the different hypotheses and mechanisms of AD.^36^, ^37^, ^38^ It is a key process that causes neuronal damage and occurs in various pathways, such as apoptosis.^36^, ^39^ So oxidative stress is confirmed as a core component of AD.^40^, ^41^ We found that Icaritin dramatically decreases the increased ROS and hydrogen peroxide in Aβ_arc_ *Drosophila*. We also found that Icaritin restores the activity of SOD, catalase, and GST damaged by Aβ_arc_ toxicity. Of course, the high level of MDA in Aβ_arc_ *Drosophila* was also downregulated by Icaritin. These results indicated that Icaritin has a powerful ability to recover the unbalanced redox homeostasis in Aβ_arc_ *Drosophila*. Moreover, oxidative stress is also one of the important reasons for apoptosis.^42^ We found that Icaritin significantly reduces the activity of Caspase‐3, which is considered the main executor of apoptosis.^43^ Moreover, immunostaining results also showed that the active Caspase‐3 positive signal of the ICT group is significantly lower than that of the WT group. It implied that inhibition of apoptosis may be an important reason why Icaritin is not limited to prolonging the lifespan of Aβ_arc_ *Drosophila*.

The brain is a highly energy‐consuming organ and relies heavily on efficient ATP production.^40^ Energy metabolism damage has always been recognized as a typical feature of AD.^40^ Fortunately, we observed that Icaritin greatly increases the level of ATP in Aβ_arc_ *Drosophila*. Adequate energy supply provides strong support for the body to combat the adverse effects of AD, such as impaired maintenance of membrane potentials and increased intracellular Ca^2+^ levels.^40^ Moreover, energy metabolism also produces a large number of intermediate products that are widely involved in various cellular biosynthetic processes, such as oxalacetic acid and α‐ketoglutaric acid.^44^ As two important intermediate products in the TCA cycle, oxalacetic acid and α‐ketoglutaric acid are precursors for the synthesis of aspartic acid and glutamic acid, respectively.^44^ That is to say, Icaritin can also help maintain a steady state of biosynthetic metabolism in AD. Moreover, the ATP production in the ICT group is significantly higher than that of the WT group. This may also be an important reason why Icaritin is not limited to prolonging the lifespan of Aβ_arc_ *Drosophila*.

Oxidative stress and energy metabolism damage are closely related in the pathological process of AD.^40^, ^45^ In brief, oxidative stress ecreases the activity of the enzymes involved in energy metabolism, leading to a precipitous fall in ATP production.^45^ Conversely, impaired energy metabolism leads to insufficient ATP production.^40^ The decrease in ATP causes an imbalance in intracellular homeostasis, such as by increasing mitochondrial membrane permeability.^40^ The result is the release of mitochondrial inclusions, including a large amount of ROS, which directly leads to oxidative stress.^40^ So oxidative stress and energy metabolism damage can fall into a mutually reinforcing vicious cycle. Incredibly, Icaritin can just break this vicious cycle and recover the unbalanced redox homeostasis and damaged energy metabolism. This may be one of the important reasons why Icaritin plays an important role in rescuing Aβ_arc_ *Drosophila*.

Transcriptome analysis provided us with a large amount of evidence to further explore the effect of Icaritin on anti‐Aβ_arc_ toxicity. We focused on verifying the genes and related pathways that were rescued by Icaritin treatment after being damaged by Aβ_arc_ toxicity, especially in oxidative stress and energy metabolism. However, the function of a large number of genes with similar expression changes should also be worth studying. Moreover, a large number of genes and related pathways that were upregulated by Aβ_arc_ toxicity and restored by Icaritin treatment have not been studied yet. There should be a lot of information worth mining. Of course, if we excluded Icaritin treatment and focused on observing the changes caused by Aβ_arc_ toxicity, more clues about the toxicity of Aβ_arc_ would be presented.

In antioxidant stress, we found that Icaritin restores the expression of Ccs (Copper/zinc superoxide dismutase), CG31028 (Copper/zinc superoxide dismutase), Cat (Catalase), Gss1 (Eukaryotic glutathione synthase), Trxr‐1 (Pyridine nucleotide‐disulphide oxidoreductase, also called thioredoxin reductase), and Prx2540 (Peroxiredoxin), which are directly involved in antioxidant stress and damaged by Aβ_arc_ toxicity. Superoxide dismutase converts superoxide ions to hydrogen peroxide.^46^ Catalase converts hydrogen peroxide to water and oxygen.^47^ Glutathione synthase catalyzes de novo glutathione synthesis, and glutathione plays crucial roles in the antioxidant defense system in neurons.^48^ The above three enzymes are the most extensively studied enzymes for antioxidant stress in AD. However, studies of thioredoxin reductase and peroxiredoxin have long overshadowed antioxidant defenses in AD.^49^, ^50^ Peroxiredoxin can scavenge ROS,^49^ thioredoxin reductase can neutralize hydrogen peroxide.^50^ The Trx‐Prx system can be used as a promising biomarker for diagnosing AD.^50^ We also found that Icaritin restores the expression of 10 genes encoding the components of GST damaged by Aβ_arc_ toxicity. GST has always been considered another important enzyme system for antioxidant stress.^51^ But its function has not been fully studied in AD. These results provided us with direct evidence of the antioxidant stress of Icaritin in AD.

In energy metabolism, glycolysis, the TCA cycle, and oxidative phosphorylation are three necessary pathways for ATP production.^44^, ^52^, ^53^, ^54^, ^55^ In glycolysis, we found that Icaritin restores the expression of Hex‐C (Hexokinase), Pfk (Phosphofructokinase), Pgk (Phosphoglycerate kinase), and Pgi (Phosphoglucose isomerase), which are directly involved in glycolysis and downregulated by Aβ_arc_ toxicity. Hexokinase and phosphofructokinase are considered key enzymes that limit the rate of glycolysis.^52^ In the TCA cycle, we found that Icaritin restores the expression of AcCoAS (Acetyl‐coenzyme A synthetase N‐terminus), Irp‐1A (aconitate hydratase), and SdhC (succinate dehydrogenase), which are directly involved in the TCA cycle and downregulated by Aβ_arc_ toxicity. Acetyl‐coenzyme A synthetase participates in the initiation of the TCA cycle and catalyzes the synthesis of acetyl‐coenzyme A from pyruvate.^44^ Aconitate hydratase is also an important component in the TCA cycle, catalyzing the process of synthesizing isocitric acid from citric acid.^44^ Succinate dehydrogenase is another important component of the TCA cycle that catalyzes the dehydrogenation of succinic acid to fumaric acid.^44^ In oxidative phosphorylation, we found that Icaritin restores the expression of a large number of genes, which encode many important components of NADH–ubiquinone oxidoreductase and ATP synthase and are downregulated by Aβ_arc_ toxicity. NADH–ubiquinone oxidoreductase is also called Complex I, which is the starting point for electrons to enter the respiratory chain and plays a crucial role in the entire oxidative phosphorylation reaction process.^54^ Its dysfunction can cause a decrease in cellular respiration by at least 40%, leading to various diseases such as AD and Parkinson's disease.^53^ ATP synthase, also known as “Complex V", is the terminal enzyme in the oxidative phosphorylation pathway.^55^ It is a huge protein complex, shaped like a mushroom that uses energy stored in the transmembrane proton gradient to drive ADP and phosphate (Pi) to synthesize ATP.^55^

The pentose phosphate pathway, which branches from glycolysis, is the main pathway of pentose metabolism for a supplement to glycolysis and the TCA cycle.^56^ We found that Aβ_arc_ toxicity damages the expression of Zw (glucose‐6‐phosphate dehydrogenase) and Pgd (6‐phosphogluconate dehydrogenase). Unfortunately, Icaritin treatment could not restore their expression. The pentose phosphate pathway also serves as a metabolic pathway for antioxidant stress, and its intermediate products provide raw materials for the synthesis of many substances.^56^ This suggested that repairing the pentose phosphate pathway may be a target against the Aβ_arc_ toxicity.

Fatty acid β‐oxidation, which acts as the principal pathway for fatty acid metabolism, is another important energy metabolism pathway.^57^ We found that Icaritin restores the expression of a large number of genes, which are directly involved in fatty acid β‐oxidation and downregulated by Aβ_arc_ toxicity. That is to say, Icaritin treatment could repair the fatty acid β‐oxidation pathway damaged by Aβ_arc_ toxicity. Although the brain mainly uses glucose as the sole energy substrate, the repaired fatty acid β‐oxidation pathway may increase ketone body formation, especially D‐β‐hydroxybutyrate, which can also serve as another energy substrate across the blood–brain barrier.^57^

The above studies suggested that Icaritin does not just target a specific point but rather systematically and comprehensively repairs the entire core process of ATP production in Aβ_arc_ *Drosophila*.

One of the main sources of ROS is the respiratory chain in the inner membrane of mitochondria.^58^ During the process of electron transfer in mitochondria, a portion of oxygen is reduced to form a superoxide anion or hydrogen peroxide.^58^ Among them, the most important is superoxide anion, which is the precursor of most ROS and is mainly produced by Complex I and III in the respiratory chain of the mitochondrial inner membrane.^58^ The central nervous system relies almost entirely on the supply of blood glucose for energy.^59^ ATP production from glucose is mainly dependent on the TCA cycle and oxidative phosphorylation in mitochondria.^60^ Therefore, ROS, which causes oxidative stress, and energy metabolism, which produces ATP, are closely related to the structural and functional integrity of mitochondria.^58^, ^59^, ^60^ In this study, we found that Icaritin can well repair the structure and function of mitochondria damaged by Aβ_arc_ toxicity. It implied that the anti‐Aβ_arc_ effect of Icaritin is at least partially achieved through the repair of mitochondria damaged by Aβ_arc_ toxicity. Otherwise, mitochondrial dysfunction drives Aβ production and Aβ plague deposition.^61^ Therefore, the repair of damaged mitochondria should be one of the mechanisms for Aβarc disappearance.

## 5 CONCLUSIONS

**FIGURE 8:**
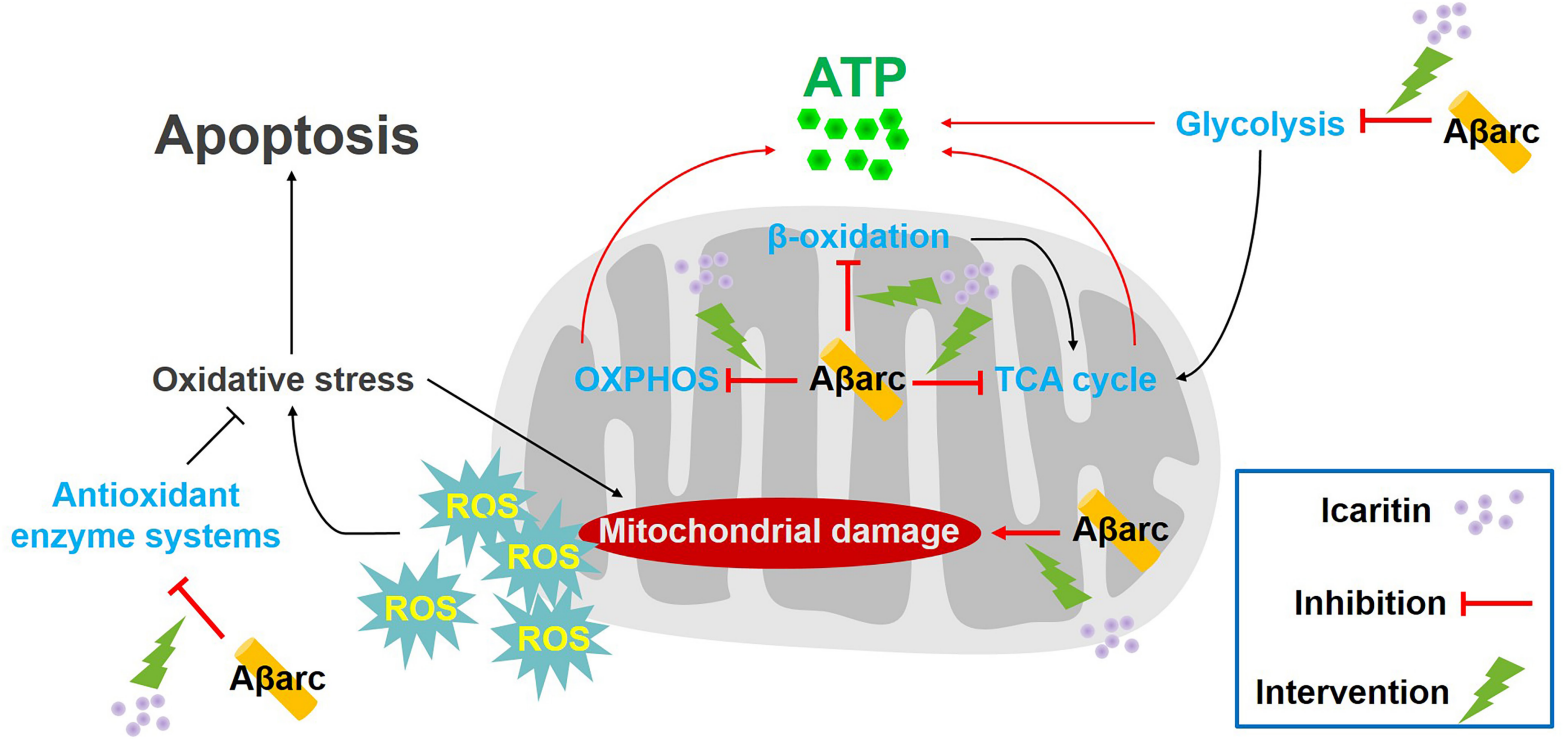
Schematic diagram of the mechanism of Icaritin against Aβ_arc_ toxicity. The blue font represents that Aβ_arc_ damages glycolysis, TCA cycle, OXPHOS, fatty acid β‐oxidation, and the antioxidant enzyme system. The green lightning symbols represent the inhibitory effect of Icaritin on the damage caused by Aβ_arc_ toxicity. OXPHOS, oxidative phosphorylation.

In summary, this study systematically elucidated for the first time that Icaritin greatly rescues Aβ_arc_ *Drosophila*, and its function is realized at least partly by restoring the mitochondria/oxidative stress/energy metabolism axis (Figure 8). This indicated that Icaritin is a highly potential anti‐AD drug and highly worthy of translating to human AD.

## AUTHOR CONTRIBUTIONS

B.X., J.T., and Q.H.L. proposed the original hypothesis, designed, and supervised the study. L.X.L., Z.W.W., and Y.F.T. performed the experiments and analyzed the data. M.Y.J., H.Y., and X.L. analyzed the data. B.X. and L.X.L. wrote and confirmed the manuscript.

## FUNDING INFORMATION

This work was supported by the Open Project Program of Guangxi Key Laboratory of Brain and Cognitive Neuroscience (GKLBCN‐20190103, GKLBCN‐20190105‐04, and GKLBCN‐202106‐02) and the Young and Middle‐aged Teachers' Basic Research Ability Improvement Project of Universities in Guangxi (2023KY0522, 2023KY0541, and 2020KY12012).

## CONFLICT OF INTEREST STATEMENT

The authors declare that they have no competing interests.

## Supporting information


Figure S1

Figure S2

Table S1

Table S2

Table S3

Table S4

Table S5

## Data Availability

The *Drosophila* brain RNA‐seq was deposited at the Sequence Read Archive with the accession code PRJNA971791. Step‐by‐step protocols are available upon request. Correspondence and requests for all other material should be addressed to Q.H.L., J.T., and B.X. Source data is provided with this paper.
