# Supplementary figures and images for: Icaritin greatly attenuates β‐amyloid‐induced toxicity in vivo
Source: CNS Neurosci Ther. 2023 Nov 21;30(4):e14527. doi: 10.1111/cns.14527 (PMC11017459; doi:10.1111/cns.14527)

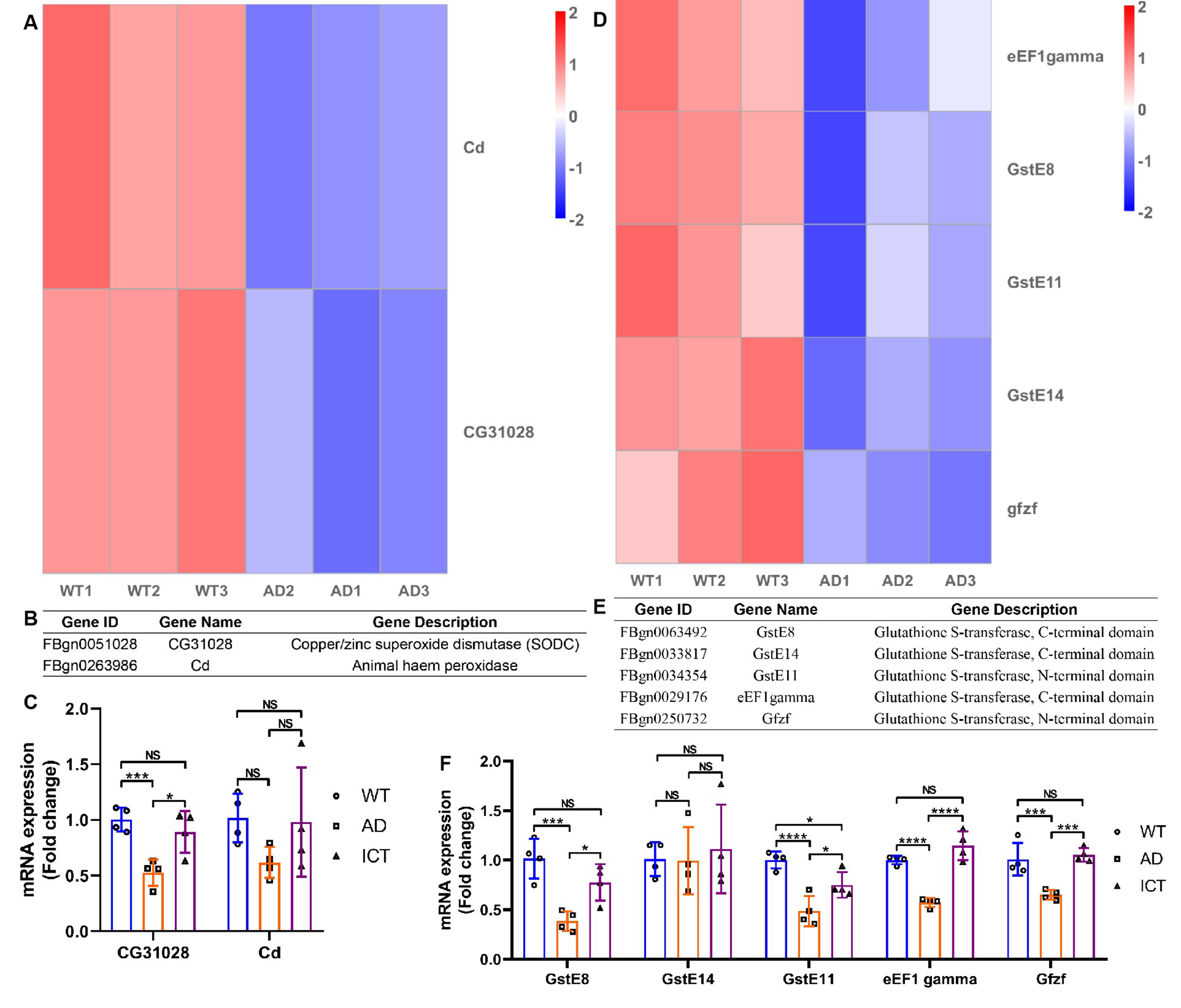

Supplement: Supplementary file 1 — Figure S1 [file CNS-30-e14527-s001.tif]

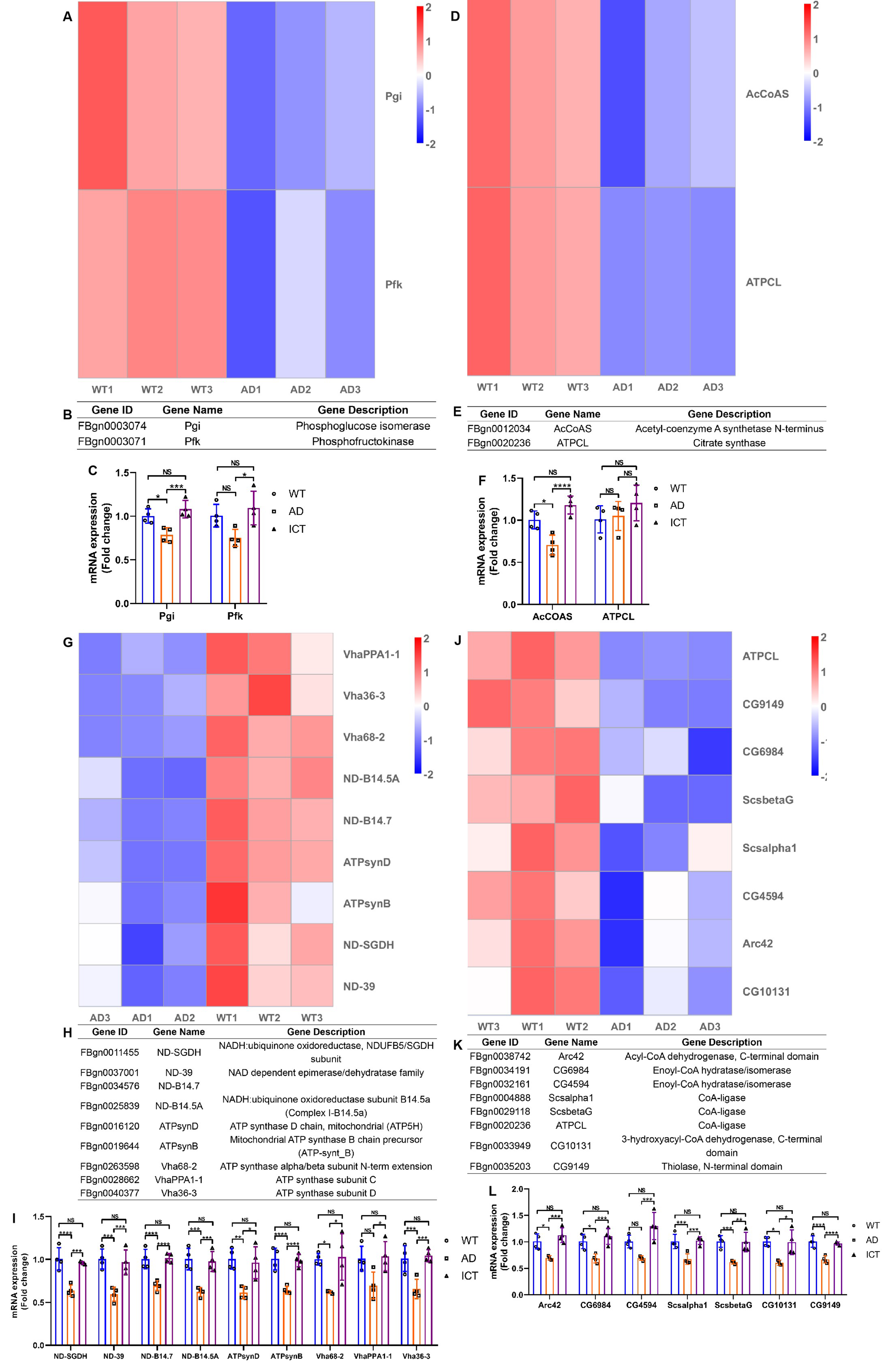

Supplement: Supplementary file 2 — Figure S2 [file CNS-30-e14527-s007.tif]
